# The role of follicular T helper cells in the humoral alloimmune response after clinical organ transplantation

**DOI:** 10.1111/tan.13671

**Published:** 2019-09-08

**Authors:** Nicole M. van Besouw, Aleixandra Mendoza Rojas, Carla C. Baan

**Affiliations:** ^1^ Department of Internal Medicine ‐ Nephrology & Transplantation, The Rotterdam Transplant Group, Erasmus MC University Medical Center Rotterdam Rotterdam the Netherlands

**Keywords:** Tfh, Tfr, Tfh1, Tfh2, Tfh17, IL‐21, rejection, ABMR

## Abstract

Over the past decade, antibody‐mediated or humoral rejection in combination with development of de novo donor‐specific antibodies (DSA) has been recognized as a distinct and common cause of transplant dysfunction and is responsible for one‐third of the failed allografts. Detailed knowledge of the mechanisms that initiate and maintain B‐cell driven antidonor reactivity is required to prevent and better treat this antidonor response in organ transplant patients. Over the past few years, it became evident that this response largely depends on the actions of both T follicular helper (Tfh) cells and the controlling counterparts, the T follicular regulatory (Tfr) cells. In this overview paper, we review the latest insights on the functions of circulating (c)Tfh cells, their subsets Tfh1, Tfh2 and Tfh17 cells, IL‐21 and Tfr cells in antibody mediated rejection (ABMR). This may offer new insights in the process to reduce de novo DSA secretion resulting in a decline in the incidence of ABMR. In addition, monitoring these cell populations could be helpful for the development of biomarkers identifying patients at risk for ABMR and provide novel therapeutic drug targets to treat ABMR.

## 1 INTRODUCTION

Antibody mediated rejection (ABMR) or “humoral” rejection is considered a major cause of early and late allograft failure.^1^, ^2^, ^3^ Interaction between T and B cells is critical for the humoral immune response. This can be protective in case of vaccination or injurious during allograft rejection after organ transplantation.

A major function of alloantigen‐activated CD4^+^ T helper cells is providing help to antigen‐activated B cells that produce antibodies. T helper cells are important in controlling of immunoglobulin class switching, somatic hypermutation of immunoglobulin variable region genes and secretion of high affinity antibodies.^4^ These events occur mainly in germinal centers (GC) in secondary lymphoid tissues. The CD4^+^ T helper cells entering the GC are recognized as T follicular helper (Tfh) cells since the year 2000.^5^, ^6^ The loss of CCR7 together with the expression of the chemokine receptor CXCR5 allows the Tfh cells to relocate from the T‐cell zones to the B‐cell follicle and cognate CXCL13 (the ligand for CXCR5) in germinal centers.^7^ Furthermore, Tfh cells express high levels of the costimulatory molecule CD40L, inducible co‐stimulator ICOS, the transcription factor Bcl6, the immune checkpoint PD‐1 (CD279), the lymphocyte activation and differentiation molecules CD84, CD200, SAP and cMAF and the main cytokine IL‐21.^5^, ^6^, ^8^, ^9^, ^10^ These factors play an important role in the activation, differentiation and survival of B cells. B cells that differentiate into plasma cells can secrete donor‐specific HLA antibodies (DSA) and may already exist prior to transplantation^11^ or develop de novo after transplantation.^12^ DSA are associated with acute and chronic allograft dysfunction resulting in progression of graft deterioration.^11^, ^12^, ^13^ Once DSA are developed, therapeutic option to clear these DSA is challenging.^14^ Therefore, alternative biomarkers to predict ABMR with DSA are necessary and could be a therapeutic target to prevent early transplant survival.

In this review we will focus on circulating Tfh cells, functional subsets of Tfh cells and the role of Tfr cells. Thereafter, we will summarize and discuss the role of circulating Tfh and Tfr cells in human organ transplantation and discuss how these cells might contribute to humoral rejection after transplantation.

## 2 CIRCULATING TFH CELLS IN PERIPHERAL BLOOD

The presence of CD4^+^CXCR5^+^ Th cells is not limited in secondary lymphoid tissues, as blood contains also this special type of cell population. Initially, these blood cells were described as recently activated T cells.^15^ Later, studies showed that blood CD4^+^CXCR5^+^ T cells have a superior capacity to CXCR5^−^ cells in inducing B cells to plasmablasts that secrete immunoglobulins.^16^, ^17^, ^18^ These reports show that blood CD4^+^CXCR5^+^ T cells contain long‐lived memory cells recognized as a circulating counterpart of Tfh cells. In addition, CXCR5^+^ T cells are more potent than CXCR5^−^ memory CD4 T cells in providing help to B cells for antibody production.^5^, ^6^, ^16^, ^19^ These cells are currently called blood memory Tfh cells or circulating Tfh (cTfh) cells.

IL‐12 plays an important role in differentiation of human Tfh cells, because it maintains the expression of ICOS and CXCR5 on naïve T cells. IL‐12 induces IL‐21 expression trough a STAT3‐dependent mechanism and is activated in human T cells exposed to IL‐12. STAT3 binds to the promotor of IL‐21 and Bcl6 genes.^20^, ^21^ STAT3 seems to have a nonredundant role in human Tfh cell differentiation. The expression of phosphorylated STAT3 on cTfh cells (CD4^+^CXCR5^+^) is positively correlated with cTfh cell frequency.^22^

Both GC Tfh and cTfh express CXCR5, while the expression of other markers is different. In contrast to GC Tfh, ICOS is only expressed in <1% of cTfh express in healthy individuals.^18^, ^23^, ^24^ It is suggested that CD4^+^CXCR5^+^CCR7^+^PD‐1^−^ICOS^−^ T cells are circulating before they will relocate to GC. After antigen reexposure these cells will be differentiated into mature Tfh with loss of CCR7 and increased expression of PD‐1 and ICOS to stimulate antibody responses.^23^ Therefore, CD4^+^CXCR5^+^CCR7^−^PD‐1^+^ICOS^+^ T cells could be identified as activated cTfh cells. This relocation can also clarify that the frequency of ICOS^+^cTfh cells were increased transiently after vaccination.^23^, ^24^ CCR7^−^PD‐1^+^ cTfh cells have a more prominent helper function than PD‐1^−^ cTfh cells, probably due to the high expression of IL‐21 and ICOS. Recently, La Muraglia et al^25^ showed that the increase in activated ICOS^+^PD‐1^+^ cTfh occurs earlier than the total cTfh (CD4^+^CXCR5^+^) and even precedes the generation of DSA in a murine transplant model.

Because in literature cTfh were differentially defined according to their phenotype, below the phenotype will be quoted in brackets.

## 3 FUNCTIONAL SUBTYPES OF TFH CELLS

Th1, Th2 and Th17 cells all have signature cytokines that are responsible for their function and express their specific transcriptional regulators, T‐bet, GATA3 and RORγt, respectively.^26^ Chemokine receptors could dissect T‐cell subsets according to their migratory capacity. CXCR3 represents differentiated Th1 cells, CCR4 differentiated Th2 cells and CCR6 differentiated Th17 cells.^27^ These three subsets can also be classified within cTfh cells: Tfh1 (CD4^+^CXCR5^+^CXCR3^+^CCR6^−^), Tfh2 (CD4^+^CXCR5^+^CXCR3^−^CCR6^−^) and Tfh17 (CD4^+^CXCR5^+^CXCR3^−^CCR6^+^). These cells produce their specific cytokines IFN‐γ (Tfh1), IL‐4 (Tfh2) and IL‐17 (Tfh17).^16^ These subsets have a different capacity to regulate humoral immunity. Only Tfh2 and Tfh17 cells induce naïve B cells to produce immunoglobulins via IL‐21. Tfh2 cells promote IgG and IgE secretion and Tfh17 promote IgG and IgA secretion.^16^ Bentibibel et al^24^ showed that Tfh1 cells can induce memory B cells, but not naïve B cells, to differentiate into plasma cells.

The biological significance of Tfh subsets is mostly reported in autoimmunity. The frequency of Tfh2 cells is increased in patients with active SLE disease, while the Tfh1 decreased, accompanied with high IgG levels in autoantibodies patient's sera. The proportion of Tfh17 cells is not associated with disease activity.^28^ While in juvenile dermatomyositis, idiopathic inflammatory myopathy, Guillain‐Barré syndrome and rheumatoid arthritis increased numbers of Tfh2 and Tfh17, and not Tfh1, were found.^16^, ^29^, ^30^, ^31^ Mainly, the Tfh2 cells were increased in IgG4‐related disease^32^ and Tfh17 cells are associated with disease severity in psoriasis and Hashimoto's thyroiditis.^33^, ^34^

## 4 T FOLLICULAR REGULATORY CELLS

Conventional FoxP3^+^ regulatory T cells suppress the activation and proliferation of effector T cells and are critical to prevent autoimmunity and may prevent rejection in solid organ transplantation.^35^ Due to the plasticity of helper T cells, Tregs can turn on Bcl6 and can express the follicular homing receptor CXCR5, resulting in Tfr phenotype.^36^, ^37^ Only a subset of Treg, 10% to 15%, can inhibit Tfh cells in murine and human lymphoid tissue.^36^, ^37^ Tfr cells share phenotypic characteristics of both conventional FoxP3^+^ Tregs and Tfh cells by expressing FoxP3, Bcl6, CXCR5, PD‐1, SAP and CD28. Both Tfh and Tfr cells co‐localize in germinal centers. Tfr cells control the germinal center reaction by limiting the numbers of Tfh cells, their cytokine production and subsequent the humoral response.^36^, ^37^, ^38^ Tfr cells are mainly induced by exposure to self‐antigen to prevent autoimmunity, because defects in Tfr cells lead to spontaneous GC formation and humoral autoimmunity.^39^

Remarkably, the T‐cell receptor repertoire of Tfr cells resembles that of Treg and differs from the repertoire of Tfh cells.^40^ This suggests, that Tfh cells promote humoral responses to nonself antigens, while Tfr cells inhibit the forming of autoantibody‐mediated autoimmunity and are also able to regulate nonantigen‐specific clones.

In the last decade, it is described that the Tfr/Tfh ratio may be a marker for the human humoral immune response. The Tfr/Tfh ratio was inversely correlated with the clinical severity of myasthenia gravis,^41^ primary biliary cholangitis^42^ and rheumatoid arthritis,^43^ and increased ratios were reported in patients with ankylosing spondylitis,^44^ Hashimoto's thyroiditis.^45^

The importance of Tregs for the control of autoimmunity and their role in transplantation has been described since two decades. However, data about the role of Tfr cells after transplantation in literature are scarce.

## 5 CIRCULATING TFH AND TFR CELLS IN ORGAN TRANSPLANTATION

Tfh cells accumulate more in lymph nodes removed during kidney transplantation compared with corresponding blood cTfh taken prior to transplantation.^46^ The percentage of cTfh cells (CD4^+^CCR5^+^) is strongly correlated with the percentage of lymph node Tfh cells.^46^ Tfh cells in the lymph nodes expressed significantly more ICOS and PD‐1 than their cTfh counterparts.^46^ Before and 3 months after kidney transplantation the percentage of cTfh cells (CD4^+^CXCR5^+^) is comparable, while their IL‐21 production was decreased after transplantation.^47^ In addition, the percentage of cTfh was higher in patients with preexistent DSA.^47^ However, Cano‐Romero et al^48^ found that the number of cTfh (CD4^+^CXCR5^+^CCR7^low^PD‐1^+^) increased after transplantation. These authors also found that patients with previous exposure to alloantigens showed a higher Tfh frequency prior to transplantation than first transplant recipients.

Before and after liver transplantation, the cTfh (CD4^+^CXCR5^+^) and cTfh17 remained stable in time. Nevertheless, the cTfh1 cells were reduced at 1 week and 1 month after transplantation compared with before liver transplantation, and the cTfh2 cells increased at 1 week and decreased to similar levels as before transplantation at 1 month posttransplant.^49^

### 5.1 Graft rejection and tolerance

Pretransplant cTfh (CD4^+^CXCR5^+^CCR7^−^PD‐1^+^) were increased in patients with a previous graft or who received blood transfusions compared with those who did not, and was higher in patients who developed rejection^48^ (Table 1). Zhang et al^50^ studied the different subsets of cTfh and rejection. These authors showed that the proportion of Tfh2 was increased and Tfh17 was decreased in patients with acute rejection compared with those without rejection. The proportion of Tfh1 cells was comparable. IL‐21 is the most important cytokine of human Tfh cell differentiation and also contributes to antibody production.^57^ The IL‐21 serum levels are higher in patients with rejection after liver transplantation than without rejection.^50^ The highest IL‐21 mRNA expression was found in heart transplant recipients undergoing rejection compared with those free from rejection.^51^ In renal transplant biopsies, IL‐21 and Bcl6 positive cells were only observed during rejection.^52^ In addition, patients who developed DSA after kidney transplantation had higher pretransplant IL‐21 plasma concentrations and more IL‐21^+^ cTfh (CD4^+^CD45RO^+^CXCR5^+^) than patients who did not develop DSA.^58^ We also showed that higher numbers of circulating donor‐reactive IL‐21 producing cells were found pretransplant in patients who had anti‐HLA antibodies compared with those without antibodies.^53^ Furthermore, high numbers of pretransplant donor‐reactive IL‐21 producing cells were associated with early rejection episodes. Moreover, high numbers of donor‐reactive IL‐21 producing cells at 6 months after transplantation correlated with late rejections.^53^

**Table 1:** Tfh cells and IL‐21 in relation to human allograft rejection and tolerance

Authors	Blood sampling (after transplantation [Tx])	Patient numbers	Cell type	Relation with rejection
Cano‐Romero et al^48^	Pre kidney Tx: acute rejection vs no rejection	18 vs 188	CD4^+^CXCR5^+^CCR7^−^PD‐1^+^	Higher
Zhang et al^50^	Liver Tx: during acute rejection vs no rejection	12 vs 20	%Tfh1 %Tfh2 %Tfh17 serum IL‐21	No Higher Lower Higher
Baan et al^51^	Heart Tx: during acute rejection vs no rejection	13 vs 44	IL‐21 in biopsy	Higher
de Leur et al^52^	Kidney Tx: during rejection	15	IL‐21^+^Bcl‐6^+^ cells in biopsy	Present
van Besouw et al^53^	Pre kidney Tx: early acute rejection vs no rejection 6 months: late rejection vs no rejection	15 vs 20 13 vs 33	Number of donor‐reactive IL‐21 producing PBMC Number of donor‐reactive IL‐21 producing PBMC	Higher Higher
Shi et al^54^	1‐3 years post kidney Tx: chronic AMBR vs no ABMR	24 vs 18	CD4^+^CXCR5^+^ CD4^+^CXCR5^+^PD‐1^+^ CD4^+^CXCR5^+^ICOS^+^ serum IL‐21	Higher Lower Comparable Comparable
Chen et al^55^	Kidney Tx: chronic ABMR vs no ABMR	40 vs 48	%CD4^+^CXCR5^+^ICOS^+^ %Tfh17%Tfh2 CD4^+^CXCR5^+^ in biopsy CD4^+^CXCR5^+^FoxP3^+^ in biopsy	Comparable Higher Higher Comparable Lower
Chenouard et al^56^	Kidney Tx: tolerance (Tol) vs stable graft function	8 vs 14	%CD4^+^CD45RA^−^CXCR5^+^ %CD4^+^CD45RA^−^CXCR5^+^PD‐1^+^ICOS1^+^ Number CD4^+^CD45RA^−^CXCR5^+^ Number CD4^+^CD45RA^−^CXCR5^+^PD‐1^+^ICOS1^+^	Tol: lower Tol: lower Comparable Comparable

Renal transplant patients with signs of chronic rejection had a significantly higher percentage of cTfh cells (CD4^+^CXCR5^+^) compared with stable patients, while PD‐1 was downregulated in cTfh cells from patients with chronic rejection. ICOS expression within Tfh cells and serum IL‐21 were comparable between these patients.^54^ Although, Chen et al^55^ showed that the percentage of cTfh cells (CD4^+^CXCR5^+^ICOS^+^) in patients with and without chronic renal allograft dysfunction (CRAD) were comparable, while the proportion Tfh17 (CD4^+^CXCR5^+^IL‐17^+^CCR3^−^CCR6^+^) and Tfh2 (CD4^+^CXCR5^+^IL‐4^+^CCR3^−^CCR6^−^) were higher in patients with CRAD. However, they found comparable numbers of Tfh cells (CD4^+^CXCR5^+^) and lower numbers of Tfr cell (CD4^+^CXCR5^+^FoxP3^+^) in biopsies from patients with ABMR compared with those without ABMR.^55^

Tolerant patients had a lower percentage cTfh (CD4^+^CD45RA^−^CXCR5^+^) than stable renal transplant patients using immunosuppression. Also the activation molecules PD‐1 and ICOS were lower in the tolerant patients.^56^ The absolute numbers of cTfh were comparable between the two groups. In contrast to the stable patients, the cTfh cells of tolerant patients failed to produce IL‐21 and could not induce B‐cell IgG production preventing de novo DSA production.

### 5.2 Immunosuppression after transplantation

In contrast to basiliximab (anti‐CD25 monoclonal antibody) induction therapy, anti‐thymocyte globuline (ATG) induction depleted cTfh (CD3^+^CD4^+^CD45RO^+^CCR5^+^) in kidney transplant recipients.^58^ The absolute number of cTfh was significantly lower from 1 month to 1 year posttransplantation in patients receiving ATG compared with those with basiliximab, while the percentage remain unchanged. This drop in Tfh cells was confirmed by Cano‐Romero et al.^48^ Patients treated with ATG had lower cTfh numbers than patients treated with basiliximab even at 6 months after transplantation. These cTfh were higher in patients who developed de novo DSA compared with the unsensitized patients. ATG induction therapy skewed the cTfh cells to the Th1, effector memory phenotype (CXCR5^+^CXCR3^+^CD45RO^+^CD62L^−^) and elevated PD‐1 expression compared with basiliximab.^58^ Patients with DSA had an increased Tfh/Treg ratio (Treg: CD127^−^FoxP3^+^) compared with stable patients.^58^

To allow ABO or HLA incompatible kidney transplantation often rituximab (anti‐CD20 monoclonal antibody) therapy is given to reduce antibody titers and depletion of circulating B cells. Rituximab in combination with tacrolimus and mycophenate mofetil removes circulating naïve B cells and not memory cells, and lymph node B cells in GC are also depleted. However, Tfh and Tfr cells are still present.^59^ Apparently, these cells do not require the GC for maintenance.

Kidney transplant recipients treated with tacrolimus have higher numbers of CD4^+^CCR5^+^ and CD4^+^CXCR5^+^PD‐1^+^ cTfh cells than patients treated with sirolimus, while CD4^+^CCR5^+^ICOS^+^ cTfh could not discriminate the patient groups.^60^ Also phosphorylated STAT3 within cTfh (CD4^+^CXCR5^+^) is higher in the tacrolimus patients than the sirolimus‐treated patients. Both the proportion and absolute cell number of cTfh (CD4^+^CXCR5^+^PD‐1^+^), activated cTfh cells (CD4^+^CXCR5^+^PD‐1^++^), Tfh1, Tfh2 and Tfh17 cells were reduced in tacrolimus‐treated patients compared with untreated patients prior to transplantation, while the conventional Th1 cells, Treg and Tfr were comparable.^61^ When tacrolimus was added in vitro, the Tfh generation (CD4^+^CRCR5^+^) was only minimally (7%) inhibited and the CD4^+^CXCR5^+^PD‐1^+^ Tfh cells were partially (48%) decreased.^62^ Tacrolimus could inhibit 50% of the donor antigen‐driven plasmablast formation.^62^ In the lymph nodes, only Tfh cells (CD4^+^CXCR5^+^PD‐1^+^) were reduced after tacrolimus treatment, and PD‐1^++^Tfh cells and Bcl6^+^Tfh cells could not discriminate the two groups of patients. Dahdal et al^63^ also showed that immunosuppression reduced the number of Tfh1 and Tfh2, but not Tfh17, cells compared with healthy individuals.

The costimulatory signal inhibitor belatacept binds CD80 and CD86 on antigen‐presenting cells (APC) and could prevent de novo DSA formation and ABMR in a nonhuman kidney transplant model by inhibition of Tfh cells in lymph nodes.^64^ Also in kidney transplant recipients treated with belatacept de novo DSA were significantly lower than cyclosporine‐treated patients,^65^ due to reduced proportion of cTfh cells (CXCR5^+^CD45RA^−^ and CXCR5^+^CD45RA^−^PD1^+^ICOS^+^).^66^ These authors showed that in vitro addition of belatacept reduced plasmablast formation and immunoglobulin production.^66^ In an earlier study was shown that belatacept could only partly suppress donor‐antigen driven plasmablast formation and was comparable with tacrolimus.^62^ Apparently, these conflicting results studying the Tfh B‐cell interaction of belatacept require further investigation.

## 6 CONCLUSION

The importance of Tfh and Tfr cells is mainly reported in B‐cell‐mediated autoimmune disease. In organ transplant recipients, studies on cTfh cells and their subsets Tfh1, Tfh2 and Tfh17 are limited. Studies measuring Tfr cells in transplantation are even more scarce. From literature it is clear that cTfh cells play a role in DSA formation. Moreover, the role of IL‐21 and a higher percentage of cTh cells during rejection and lower percentage cTfh in tolerant patients implies the importance of monitoring these special cells. However, the role of Tfr cells in tempering DSA formation to prevent ABMR is not yet elucidated. Although, avoiding de novo DSA formation will increase transplant survival and suggests the importance of studying the Tfh/Tfr ratio. Therefore, it will be attractive to monitor the Tfh/Tfr ratio in combination with IL‐21 in the first year after transplantation as potential biomarkers to identify patients at risk for ABMR. In addition, targeting the molecules leading to alloantigen activation and IL‐21 secretion of Tfh cells will be a relevant approach to prevent B cell differentiation and subsequent production of de novo DSA resulting in a decrease in the incidence of ABMR. Furthermore, understanding the role of donor‐specific Tfh and Tfr cells in the humoral immune response should be performed including their specific proteome profile in combination with different immunosuppressive medication. This will unravel the biological function of these cells in the organ transplant setting, and lead to improved therapeutic strategies with the ultimate goal of personalized immunosuppressive medication in patients at risk for ABMR.

In summary, there is a requirement for a robust biomarker for identification of patients at risk for de novo DSA formation and development of ABMR. In addition, the knowledge of the biological mechanisms underlying ABMR could be a step forward to improve therapeutic regimens and to develop novel therapeutic strategies to both prevent and treat ABMR resulting in a higher allograft survival.

## CONFLICT OF INTEREST

The authors have declared no conflicting interests.
